# Compound *Bacillus* alleviates diarrhea by regulating gut microbes, metabolites, and inflammatory responses in pet cats

**DOI:** 10.1186/s42523-023-00270-8

**Published:** 2023-10-10

**Authors:** Fei Wang, Xiaoying Mei, Qi Wang, Pengwei Zhao, Yuanhao Zhou, Li Tang, Baikui Wang, Shujie Xu, Xiang Li, Qian Jin, Yingping Xiao, Weifen Li

**Affiliations:** 1 Key Laboratory of Animal Molecular Nutrition of Education of Ministry, National Engineering Laboratory of Biological Feed Safety and Pollution Prevention and Control, Key Laboratory of Animal Feed and Nutrition of Zhejiang Province, Institute of Animal Nutrition and Feed Sciences, College of Animal Sciences, Zhejiang University, Hangzhou, 310058 China; 2 Hangzhou Wangmiao Biotechnology Co., LTD, Hangzhou, 311112 China; 3 State Key Laboratory for Managing Biotic and Chemical Threats to the Quality and Safety of Agro-Products, Institute of Agro-Product Safety and Nutrition, Zhejiang Academy of Agricultural Sciences, Hangzhou, 310021 China

**Keywords:** *Bacillus*, Pet cats, Diarrhea, Microbiome, Fungi, Metabolomics

## Abstract

**Background:**

Pet cats frequently have diarrhea in their daily life. *Bacillus* has a protective role that has crucial beneficial functions on intestinal homeostasis. The aim of this research was to investigate the effects of the compound *Bacillus* on the prevention of diarrhea, microbiota and metabolism in pet cats. A total of 20 pet cats (1–2 years old, 3.91 ± 0.92 kg) were randomly divided into two groups and fed with a basal diet (Control group), or a basal diet supplemented with 3 × 10^9^ CFU/kg compound *Bacillus* (Probiotics group). The experiment lasted 33 days.

**Results:**

Results showed that the compound *Bacillus* significantly reduced the rate of soft stools and diarrhea in pet cats compared with the control group (*P* < 0.05, n = 10). Meanwhile, compared with the control group, the probiotics group significantly decreased the content of IL-1β and IL-6 and significantly increased IL-10 (*P* < 0.05, n = 6) in the serum. In addition, feeding probiotics significantly increased the abundance of p_Patescibacter and g_*Plectosphaerella*, decreased the abundance of p_Firmicutes, p_Gemmatimonadetes, g_*Ruminococcaceae_UCG-005*, g_*Ascochytahe* and g_*Saccharomyces* in the feces of the pet cats (*P* < 0.05, n = 6). And it also can significantly increase the content of total SCFAs, acetic acid and butyric acid in the feces (*P* < 0.05, n = 6). The fecal and serum metabolomics analyses revealed that most fecal and serum compounds were involved in metabolism, particularly in chemical structure transformation maps and amino acid metabolism. Also, eugenitol and methyl sulfate were the most significantly increased serum metabolites, and log_2_FC were 38.73 and 37.12, respectively. Pearson’s correlation analysis showed that changes in serum metabolism and fecal microbiota were closely related to immune factors. There was also a strong correlation between serum metabolites and microbiota composition.

**Conclusions:**

The results of this research highlight the potential of the compound *Bacillus* as a dietary supplement to alleviate diarrhea in pet cats.

**Supplementary Information:**

The online version contains supplementary material available at 10.1186/s42523-023-00270-8.

## Introduction

With the development of the economy, people's living standards are getting better and better, more people are keeping pets and their interest in the health and well-being of animals has increased dramatically [1]. Diarrhea diseases among pet cats are frequent in daily feeding life, due to animal exposition on stress, diet change, and spread of bacterial, viral, and parasitic agents [2]. At present, studies on client-owned pets have shown that probiotics [2], functional amino acids [3, 4], plant extracts [5, 6], seaweed powder [7], vitamins [8, 9], trace elements [10] have certain immunomodulatory effects.

In 2002, the Food and Agriculture Organization of the United Nations (FAO) and the World Health Organization (WHO) defined probiotics as "living microorganisms that, when offered in sufficient quantities, provide the host with health benefits". Probiotics have many beneficial effects, such as inhibiting the colonization of pathogenic bacteria, maintaining the homeostasis of intestinal flora, developing the intestinal mucosal structure, promoting the digestion and absorption of nutrients, strengthening the immune function of the body, and preventing animal stress and diarrhea. Our previous studies have shown that BaSC06 is an oral probiotic with a number of proven beneficial effects. For example, BaSC06 enhances intestinal tight junction expression [11], improves immune function [12] and intestinal microbiome composition [13] in broilers. Studies in pigs showed that BaSC06 could improve growth performance, antioxidant and immune functions [14], and induce AKT-FOXO-mediated autophagy, thereby reducing oxidative stress-induced IPEC-J2 cell apoptosis and cell damage [15]. We also found that BaSC06 modified microbiota can induce M2 polarization in macrophages, thereby ameliorating salmonella inflammation in mice [16]. In addition, we have found that B10 can improve the intestinal microflora of broilers [17] and reduce weight gain in diet-induced obese mice by improving lipid metabolism and oxidative stress state [18].

Although previous studies have shown that probiotics can improve immune function and prevent diarrhea in pet cats [19], the underlying mechanisms have not been well elucidated. The objective of this study is to investigate the effects of probiotics on intestinal microbiota and metabolites in pet cats by integrating microbiome and metabolomics methods, in order to reveal the mechanism of probiotics in preventing diarrhea and improving immune function in pet cats.

## Materials and methods

### Animal ethics

All experimental procedures were conducted in accordance with the Animal Welfare Committee guidelines and the experimental protocol was approved by the Animal Care and Use Committee of Zhejiang University (Hangzhou, China).

### Experimental material

Compound probiotics preparation (containing *Bacillus amyloliquefaciens* SC06 (BaSC06) and *Bacillus subtilis* 10 (B10), both isolated and preserved by our laboratory) was prepared and provided by our laboratory. The concentration of BaSC06 and B10 solution were about 1 × 10^10^ CFU/ml and 1.1 × 10^10^ CFU/ml, and the two strains were mixed in a ratio of 1:1. The basal diet and the experimental diet supplemented with compound probiotics were prepared with using after extrusion process with the coating process by Hangzhou Wangmiao Biological Technology Co., LTD.

### Animals and experimental design

**Fig. 1:**
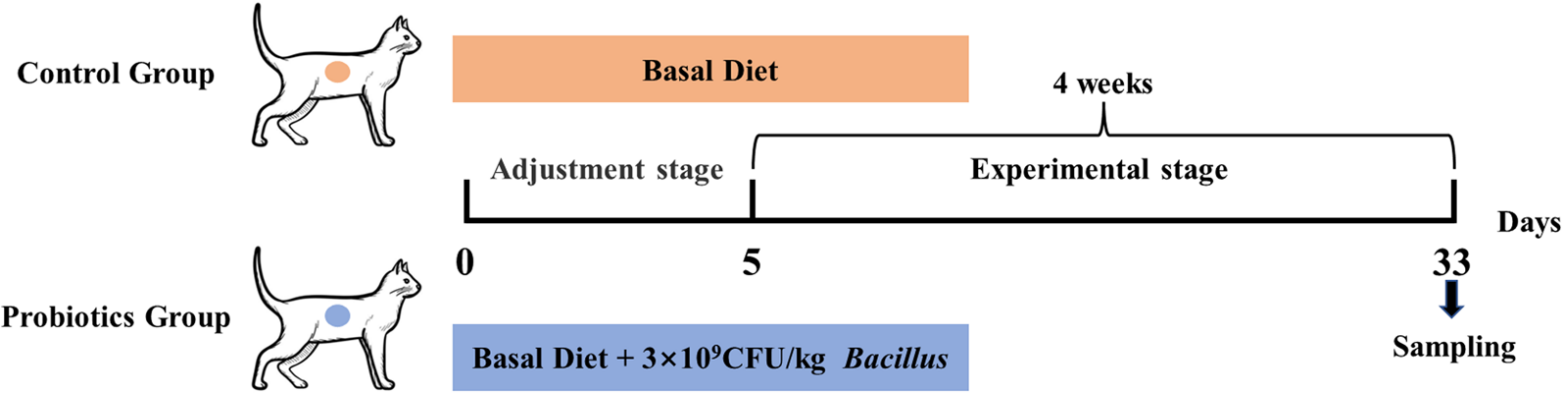
Schematic diagram of experimental design. Control group: fed with a basal diet, Probiotics group: fed with a basal diet and 3 × 10^9^ CFU/kg compound *Bacillus*

Twenty ragdoll cats were selected according to the following criteria: 1–2 years old with an average body weight of 3.91 ± 0.92 kg, healthy with no previous medical history. They were randomly divided into two groups with ten replicates per group and one cat per replicate. Prior to the trial, the necessary immunization and deworming treatments were performed, and no drugs (such as antibiotics) that would alter the intestinal microflora were received within 1 month prior to the trial. Shovel excrement once a day in the morning and at night, change the cat litter once a week, clean and disinfect the cat house every day, keep the pet enclosure clean. The control group was fed a basal diet, and the experimfental group was fed an experimental diet containing 3 × 10^9^ CFU/kg compound *Bacillus*. The basal diet met the nutritional requirements of AAFCO (2017), and its composition and nutritional levels were shown in Additional file 1 (Table S1). The cats were allowed to drink and eat freely throughout the experiment. The experiment lasted 33 days, including 5 days for pre-test and 28 days for formal test (Fig. 1).

### Fecal sample collection and analysis

From the first day of the formal experiment after the end of the pre-experiment and continuing for four weeks, with minor modifications to the stool scoring criteria of Fusi et al. [20], the fecal scores (FS) were assessed daily according to Additional file 1 (Table S2). The diarrhea rate and soft stool rate of each group were calculated by the following formula: Diarrhea rate (%) = 100 × the number of the cats had diarrhea during the trial period/the total number of fecal scores was counted during the experiment period, Soft stool rate (%) = 100 × the number of the cats had soft stools during the trial period/the total number of fecal scores was counted during the experiment period. On the last day of the experiment, six cats were randomly selected from each group to collect fresh fecal samples from cat's litter box within 15 min of defecation to a 5-ml sterile fecal collection tube, quick-frozen with liquid nitrogen, and then transferred to − 80 °C for storage for subsequent short-chain fatty acid measurement, microbiota and metabolomics analysis.

### Blood sample collection and analysis

After fasting overnight, six cats were randomly selected from each group to collect blood samples by forelimb vein and placed for 30 min, followed by centrifugation at 3500 × g at room temperature for 15 min. After centrifugation, the supernatants were taken into microcentrifuge tubes and stored at − 80 °C for further analysis. Serum interleukin (IL)-1β, IL-4, IL-6, IL-8, IL-10, IL-18 and interferon (IFN)-γ were measured using commercial ELISA kits (Jiancheng Bioengineering Institute, Nanjing, Jiangsu, China). All experiments had six replicates. Finally, an aliquot for serum metabolomics analysis was snap-frozen on liquid nitrogen and stored at − 80 °C until analysis.

### Microbiome data analysis

Genomic DNA from individual feces samples were extracted using the TGuide S96 Magnetic Soil/Stool DNA Kit (Tiangen, Beijing, China) according to the manufacturer’s instructions. The DNA concentration of the samples was measured with the Qubit dsDNA HS Assay Kit and Qubit 4.0 Fluorometer (Invitrogen, Thermo Fisher Scientific, Oregon, USA). The DNA samples were subjected to PCR amplification with primers 338F (5′-ACTCCTACGGGAGGCAGCA-3′) and 806R (5′-GGACTACHVGGGTWTCTAAT-3′) targeting the V3-V4 hypervariable region of the 16S rRNA gene, and with primers ITS1F (5′-CTTGGTCATTTAGAGGAAGTAA-3′) and ITS2 (5′-GCTGCGTTCTTCATCGATGC-3′) targeting the ITS1 region of the ITS gene. The total of PCR amplicons was purified with Agencourt AMPure XP Beads (Beckman Coulter, Indianapolis, IN) and quantified using the Qubit dsDNA HS Assay Kit and Qubit 4.0 Fluorometer (Invitrogen, Thermo Fisher Scientific, Oregon, USA). After the individual quantification step, amplicons were pooled in equal amounts. For the constructed library, use Illumina novaseq 6000 (Illumina, Santiago CA, USA) for sequencing.

According to quality of single nucleotide, raw data was primarily filtered by Trimmomatic (version 0.33). Identification and removal of primer sequences was processed by Cutadapt (version 1.9.1). PE reads obtained from previous steps were assembled by USEARCH (version 10) and followed by chimera removal using UCHIME (version 8.1). The high-quality reads generated from the above steps were used in the following analysis. Sequences with similarity ≥ 97% were clustered into the same operational taxonomic unit (OTU) by USEARCH (v10.0), and the OTUs with abundance < 0.005% were filtered. The most frequently occurring sequence was extracted as the representative sequence for each OTU and was screened for further annotation using Silva (for 16S, https://www.arb-silva.de/) and Unite (for ITS, http://unite.ut.ee/index.php) databases with the confidence threshold set to default to ≥ 0.5. The OTU taxonomy synthesis information table (out_ table) for the final analysis was obtained after removing the OTUs and tags, which are annotated as chloroplasts or mitochondria (16S amplicons), or organisms other than fungi (ITS amplicons), and could not be annotated to the kingdom level.

### Fecal and serum metabolic profiling analysis by untargeted metabolomics

Briefly, frozen stool samples stored at − 80 °C were thawed at 4 °C. Approximately 60 mg of sample was weighed and put into 2-ml round-bottom microcentrifuge tubes. Metabolites were extracted by adding 600 µl of methanol: water (1:1, v/v), and magnetic beads were added to the microcentrifuge tubes for homogenization using a homogenizer. Ultrasonic crushing was performed at a low temperature for 10 min, followed by − 20 °C for 30 min. The samples were then centrifuged at 13,000 g, 4 °C for 15 min, and 200 µl of supernatant was dried in a vacuum centrifuge. Immediately afterward, the samples were redissolved with 200 µl of 50% methanol each and vortexed for 2 min. After ultrasonic crushing for 10 min at a low temperature, the microcentrifuge tube was centrifuged again at 13,000 g, 4 °C for 15 min. Finally, the supernatant was stored in a sample injection bottle for analysis. Metabolites were obtained from the serum samples using a methanol/water (4:1, v/v) solution. The mixture was settled at − 20 °C, crushed at 50 Hz for 6 min, and ultrasonicated at 40 kHz for 30 min at 5 °C. Then, the samples were stood at − 20 °C for 30 min to precipitate the proteins. After centrifugation at 13,000 g, 4 °C for 15 min, the supernatant was transferred to sample vials for analysis.

1 μl of the separated samples were injected and detected on the LC/MS system for metabolomics analysis is composed of Waters Acquity I-Class PLUS ultra-high performance liquid tandem Waters Xevo G2-XS QT of high resolution mass spectrometer. The column used is purchased from Waters Acquity UPLC HSS T3 column (1.8 um 2.1*100 mm; Waters Corporation, Milford, MA, USA). The mobile phases containing 0.1% formic acid aqueous solution and 0.1% formic acid acetonitrile, and the flow rate was 400 μl/min. Waters Xevo G2-XS QTOF high resolution mass spectrometer can collect primary and secondary mass spectrometry data in MSe mode under the control of the acquisition software (MassLynx V4.2, Waters). In each data acquisition cycle, dual-channel data acquisition can be performed on both low collision energy and high collision energy at the same time. The low collision energy is 2 V, the high collision energy range is 10 ~ 40 V, and the scanning frequency is 0.2 s for a mass spectrum. The parameters of the ESI ion source are as follows: Capillary voltage: 2000 V (positive ion mode) or − 1500 V (negative ion mode); cone voltage: 30 V; ion source temperature: 150 °C; desolvent gas temperature 500 °C; backflush gas flow rate: 50 L/h; desolventizing gas flow rate: 800 L/h.

The raw data collected using MassLynx V4.2 is processed by Progenesis QI software for peak extraction, peak alignment and other data processing operations, based on the Progenesis QI software online METLIN database and Biomark’s self-built library for identification, and at the same time, theoretical fragment identification and mass deviation all are within 100 ppm. After normalizing the original peak area information with the total peak area, the follow-up analysis was performed. Principal component analysis and Spearman correlation analysis were used to judge the repeatability of the samples within group and the quality control samples. The identified compounds are searched for classification and pathway information in KEGG, HMDB and lipidmaps databases. According to the grouping information, calculate and compare the difference multiples, T test was used to calculate the difference significance p-value of each compound. The R language package ropls were used to perform PLS-DA modeling, and 200 times permutation tests were performed to verify the reliability of the model. The VIP value of the model was calculated using multiple cross-validation. The method of combining the difference multiple, the *P* value and the VIP value of the OPLS-DA model was adopted to screen the differential metabolites. The screening criteria are FC > 1, *P* value < 0.01 and VIP > 1. The difference metabolites of KEGG pathway enrichment significance were calculated using hypergeometric distribution test.

### Analysis of SCFAs in the fecal contents by gas chromatography

The protocol for analysis of SCFAs in the fecal contents was detected on a wet basis by gas chromatography, which conducted according to previous study described [21]. Briefly, 100 mg of fecal content was homogenized with 1 mL of sterile PBS, centrifuged at 12,000 rpm and 4 °C for 10 min. Then, 500 μl aliquot of the supernatant fluid was diluted with 100μL of 25% (w/v) metaphosphoric acid solution. The mixture was incubated at − 20 °C for 24 h, then centrifuged at 4 °C and 12,000 rpm for 10 min. Finally, the supernatant was filtered through a 0.22 μm syringe filter and injected into Shimadu GC-2030 ATF instrument for SCFAs detection. The carrier gas was N_2_ (pressure, 12.5 Mpa and flow, 18 mL min^−1^), the temperature of the injector and detector was 180 °C, and the column was gradually heated from 80 to 170 °C at a rate of 4 °C min^−1^.

### Statistical analysis

All data were recorded with Excel software (Microsoft Inc, Washington DC, USA). Microbiome data analysis methods are described in section "Microbiome data analysis" and metabolomics data analysis methods are described in "Fecal and serum metabolic profiling analysis by untargeted metabolomics" section. Other data are tested for normal distribution first. Student’s t-test will be performed when they conform to normal distribution, if not, Mann–whitney U test will be performed by SPSS software (SPSS Inc., Chicago, IL, USA). Results were expressed as means ± standard deviation (SD), and the values of *P* < 0.05 was considered to indicate a statistically significant difference and tendencies were at *P* < 0.10. Graphs were generated by GraphPad Prism 8.0 software (GraphPad Software, San Diego, CA, USA).

## Results

### Compound Bacillus decreased diarrhea rate in pet cats

**Fig. 2:**
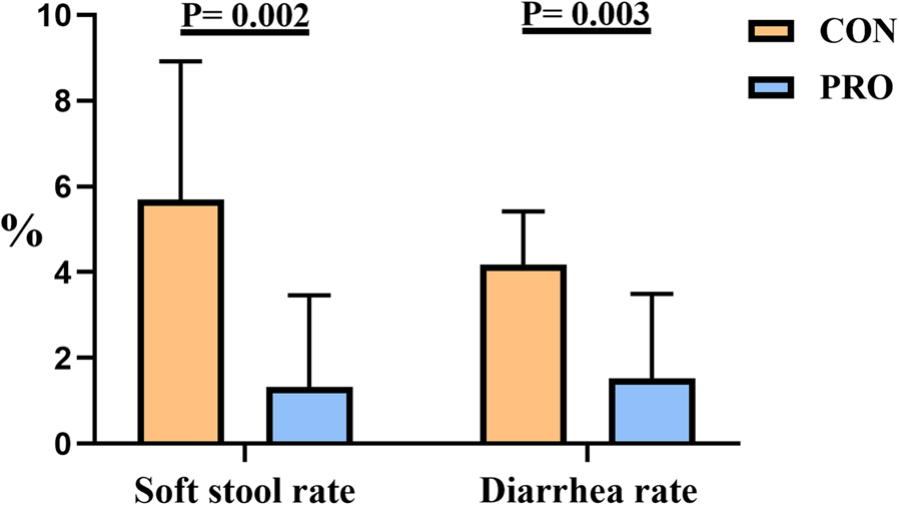
Compound *Bacillus* alleviate the rate of soft stool and diarrhea in pet cats. CON: Control group, PRO: Probiotics group. Data are expressed as mean ± SD (n = 10 per group). The differences were analysed by Student’s t-test

Compound *Bacillus* significantly reduced the rate of soft stools and diarrhea in pet cats compared with the control group (*P* < 0.05). The soft stool rates of the control group and the experimental group were 5.69% and 1.33%, and the diarrhea rates were 4.17% and 1.52%, respectively (Fig. 2).

### Compound Bacillus modulated serum cytokines levels in pet cats

**Fig. 3:**
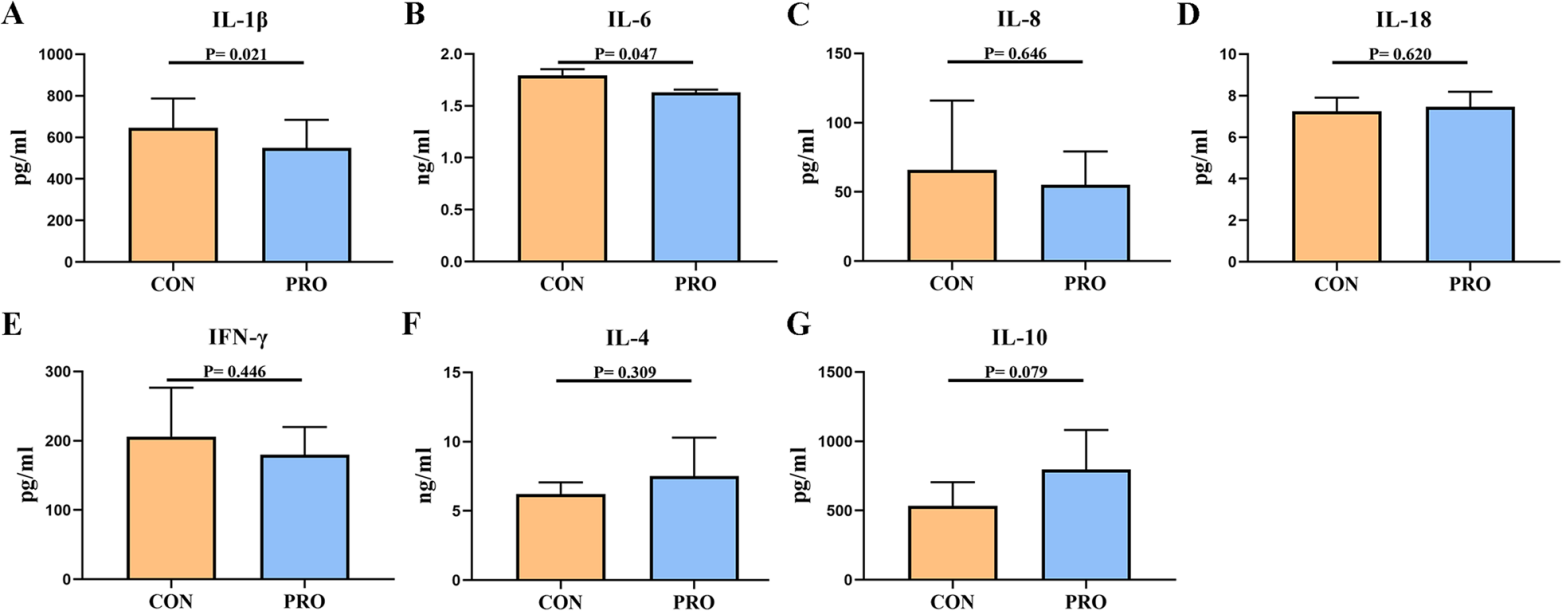
Compound *Bacillus* modulated the immune function of pet cats. **A** IL-1β, **B** IL-6, **C** IL-8, **D** IL-18, **E** IFN-γ, **F** IL-4, **G** IL-10. CON: Control group, PRO: Probiotics group. Data are expressed as mean ± SD (n = 6 per group). The differences were analysed by Student’s t-test

Probiotic treatment significantly decreased the contents of proinflammatory cytokines (IL-1β, CON: 681.16 pg/ml vs. PRO: 514.72 pg/ml, and IL-6, CON: 1.75 ng/ml vs. PRO: 1.66 ng/ml, *P* < 0.05), and increased the content of anti-inflammatory cytokines (IL-10, CON: 532.62 pg/ml vs. PRO: 797.49 pg/ml, *P* < 0.1) in serum (Fig. 3). However, there was no significant effect on other inflammatory factors (IL-8, IL-18, IFN-γ and IL-4, *P* > 0.05) in the serum (Fig. 3).

### Compound Bacillus improved fecal bacteria of diversity and composition in pet cats

**Fig. 4:**
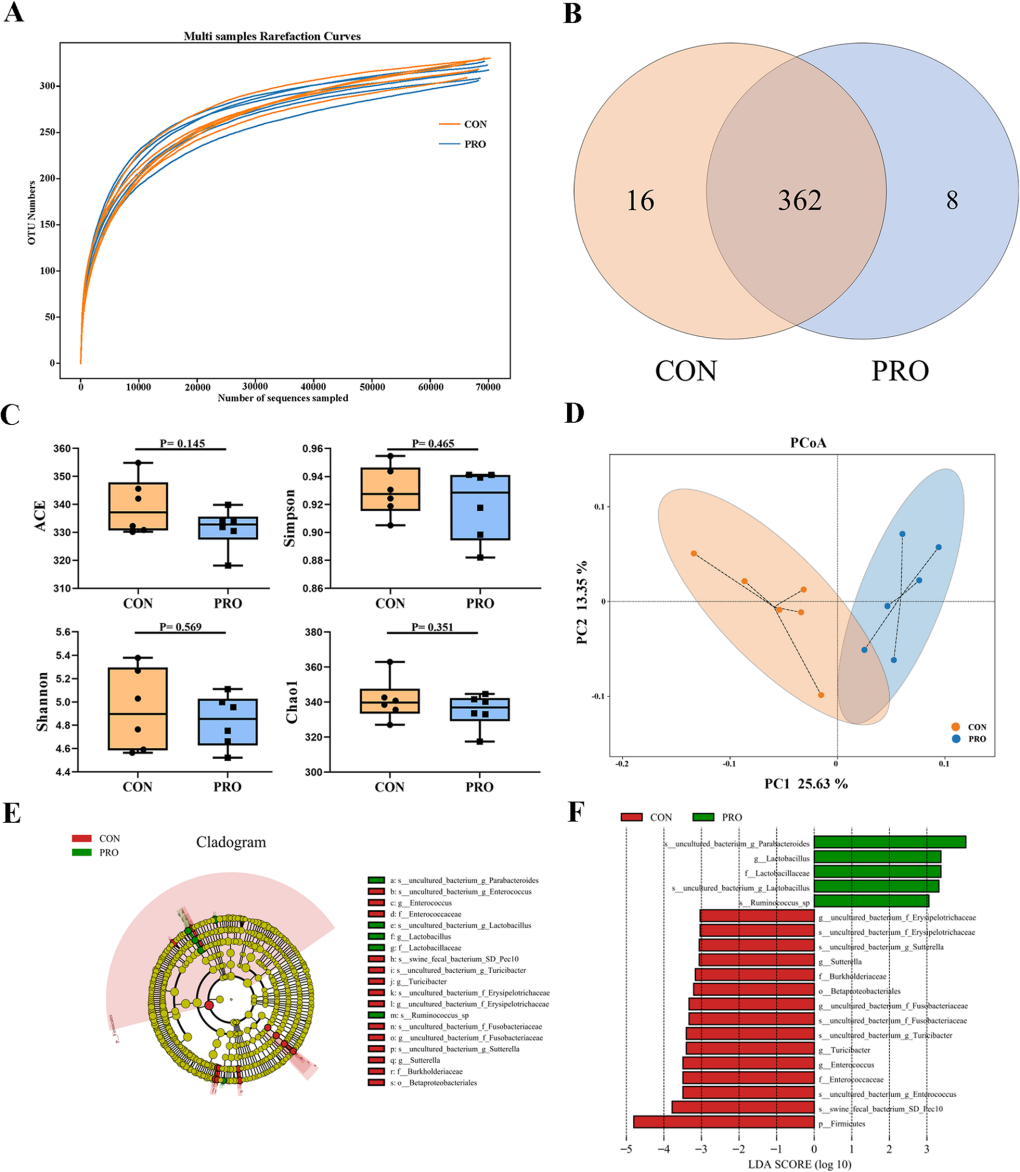
Diversity and overall composition of fecal bacteria between two groups. **A** Multi samples rarefaction curves. **B** Venn diagram presenting the operational taxonomic units (OTUs) from each group. **C** Alpha diversity (ACE, Simpson, Shannon and Chao 1). **D** Principal coordinate analysis (PCoA) based on binary_ jaccard distances. **E** The cladogram of LEfSe analysis. **F** The histogram of LEfSe analysis. CON: Control group, PRO: Probiotics group. Data are expressed as mean ± SD (n = 6 per group)

Rarefaction curve analysis of OTUs in all samples approached a plateau, indicating that the sampling depth of all samples was sufficient to capture the overall bacteria diversity (Fig. 4A). The common OTUs of the CON and PRO groups were 362, and the unique OTUs of the two groups were 16 and 8, respectively (Fig. 4B). The PRO group had no significant effect on α diversity (ACE, Simpson, Shannon and Chao 1) compared with the control group (*P* > 0.05) (Fig. 4C), but the β diversity displayed in the PCoA scatterplot indicated an obvious a significant shift of the PRO group from the CON group, with 25.63% and 13.35% variation, explained by principal components 1 and 2, respectively (Fig. 4D). LEfSe analysis (LDA > 3.0) found significant differences in fecal bacterial abundance, and there were 20 taxonomic biomarkers between the two groups (Fig. 4E, F). Probiotics treatment markedly increased the relative abundance of *Lactobacillaceae* (family) and *Lactobacillus* (genus). The CON group significantly increased the relative abundance of Firmicutes (phylum), Betaproteobacteriales (order), *Enterococcaceae* (family), *Burkholderiaceae* (family), *Enterococus* (genus), *Turicibacter* (genus) and *Sutterella* (genus).

**Fig. 5:**
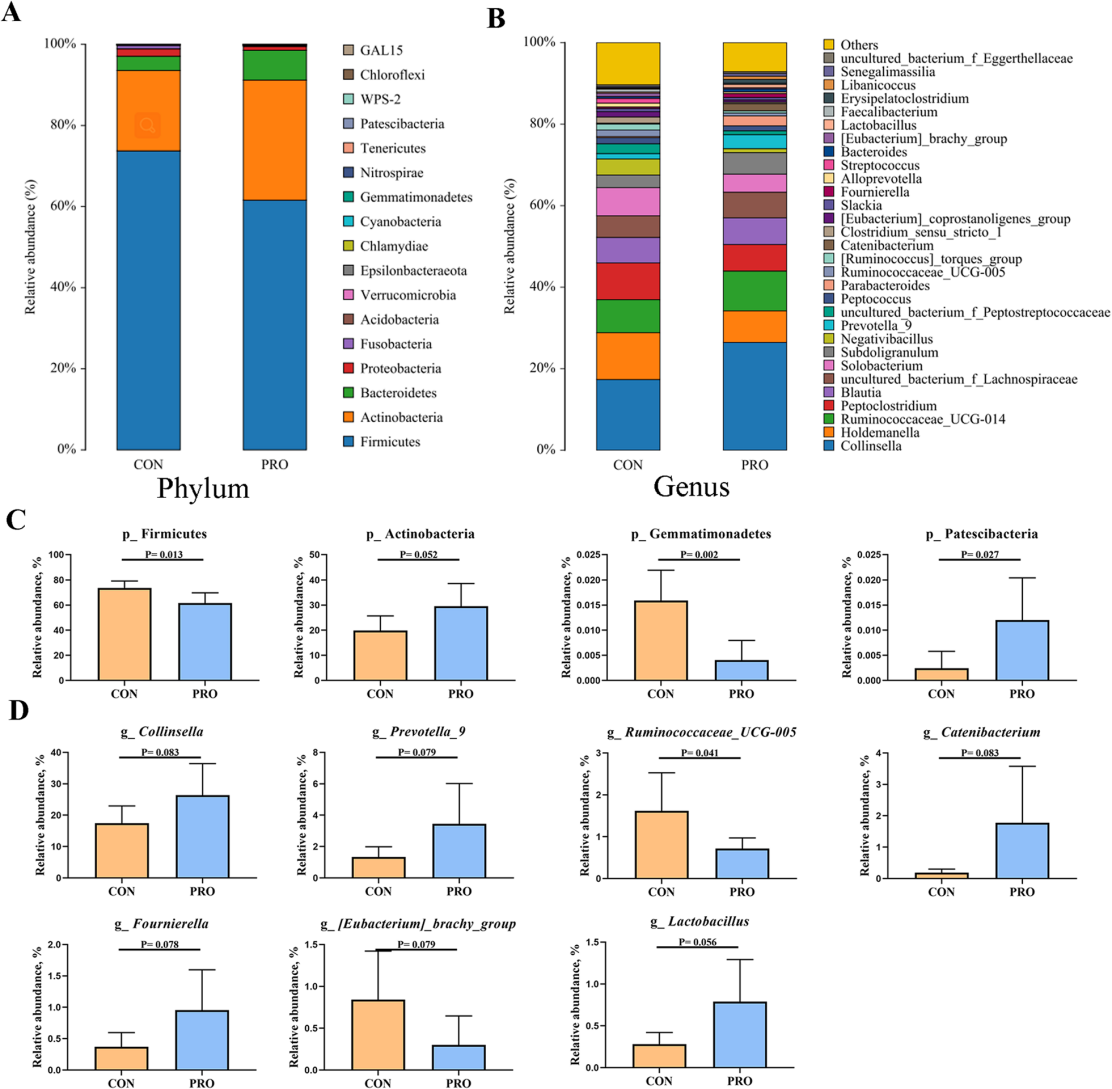
**A**, **B** Average relative abundance of bacteria species in the feces at the phylum level (**A**) and genus level (**B**). **C**, **D** Relative abundance of bacteria communities in the feces contents at the phylum level (**C**) and genus level (**D**). CON: Control group, PRO: Probiotics group. Data are expressed as mean ± SD (n = 6 per group)

The relative abundances of different phyla and genus were presented in Fig. 5. At the phylum level, Firmicutes and Actinobacteria accounted for more than 90% of the total bacteria in pet cats. Compared with the control group, the increased abundance of Patescibacteria (*P* < 0.05) and Actinobacteria (*P* < 0.1) and the decreased abundance of Firmicutes (*P* < 0.05) and Gemmatimonadetes (*P* < 0.05) were observed in the PRO group. At the genus level, probiotic *Bacillus* significantly decreased the relative abundance of *Ruminococcaceae_UCG-005* (*P* < 0.05), but increased the abundance of *Collonsella*, *Prevotella_9*, *Catenibacterium*, *Fournierella* and *Lactobacillus* (*P* < 0.1).

### Compound Bacillus improved fecal fungi of diversity and composition in pet cats

**Fig. 6:**
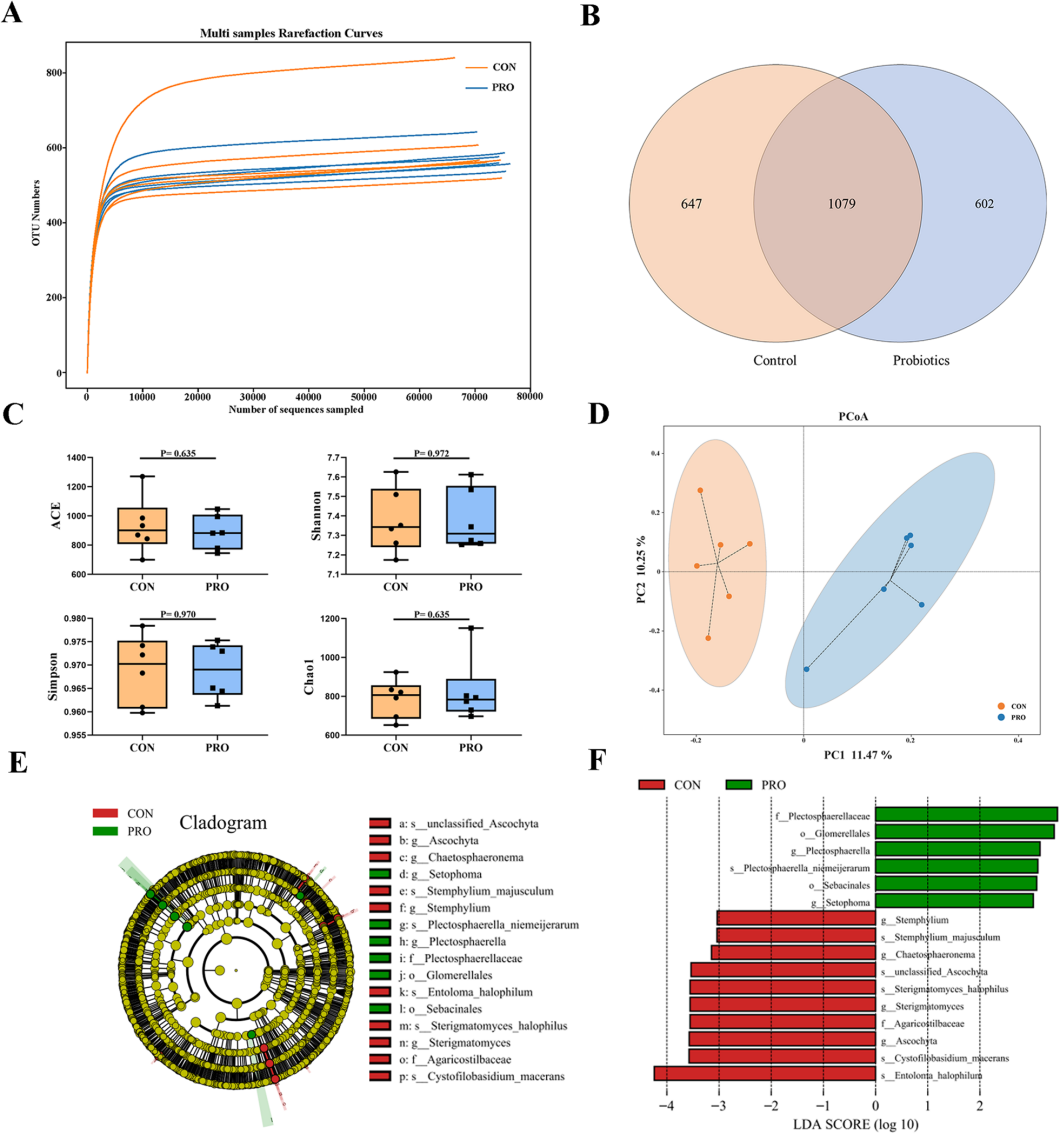
Diversity and overall composition of fecal fungus between two groups. **A** Multi samples rarefaction curves. **B** Venn diagram presenting the operational taxonomic units (OTUs) from each group. **C** Alpha diversity (ACE, Simpson, Shannon and Chao 1). **D** Principal coordinate analysis (PCoA) based on binary_jaccard distances. **E** The cladogram of LEfSe analysis. **F** The histogram of LEfSe analysis. CON: Control group, PRO: Probiotics group. Data are expressed as mean ± SD (n = 6 per group)

The sampling depth of all samples was sufficient to capture the overall fungi diversity (Fig. 6A). There were 1079 identical OTUs in the two groups, and 647 and 602 unique OTUs in the CON and PRO groups, respectively (Fig. 6B). There was no significant difference in α diversity between the two groups of fungi (*P* > 0.05) (Fig. 6C), whereas PCoA of the two groups were well separated, which is explained by principal components 1 and 2 with 11.47% and 10.25% variation, respectively (Fig. 6D).

LEfSe analysis further found that there were significant differences in the relative abundance of fungi in the fecal microbiota between the two groups (Fig. 6E, F). Fungi taxa with LDA score greater than 3.0 are selected as biomarker taxa, and we found that 16 taxa biomarkers in the two groups, including Glomerellales (order), Sebacinales (order), *Plectosphaerellaceae* (family), *Agaricostilbaceae* (family), *Plectosphaerella* (genus), *Setophoma* (genus), *Stemphylium* (genus), *Chaetosphaeronema* (genus), *Sterigmatomyces* (genus), *Ascochyta* (genus), etc.

**Fig. 7:**
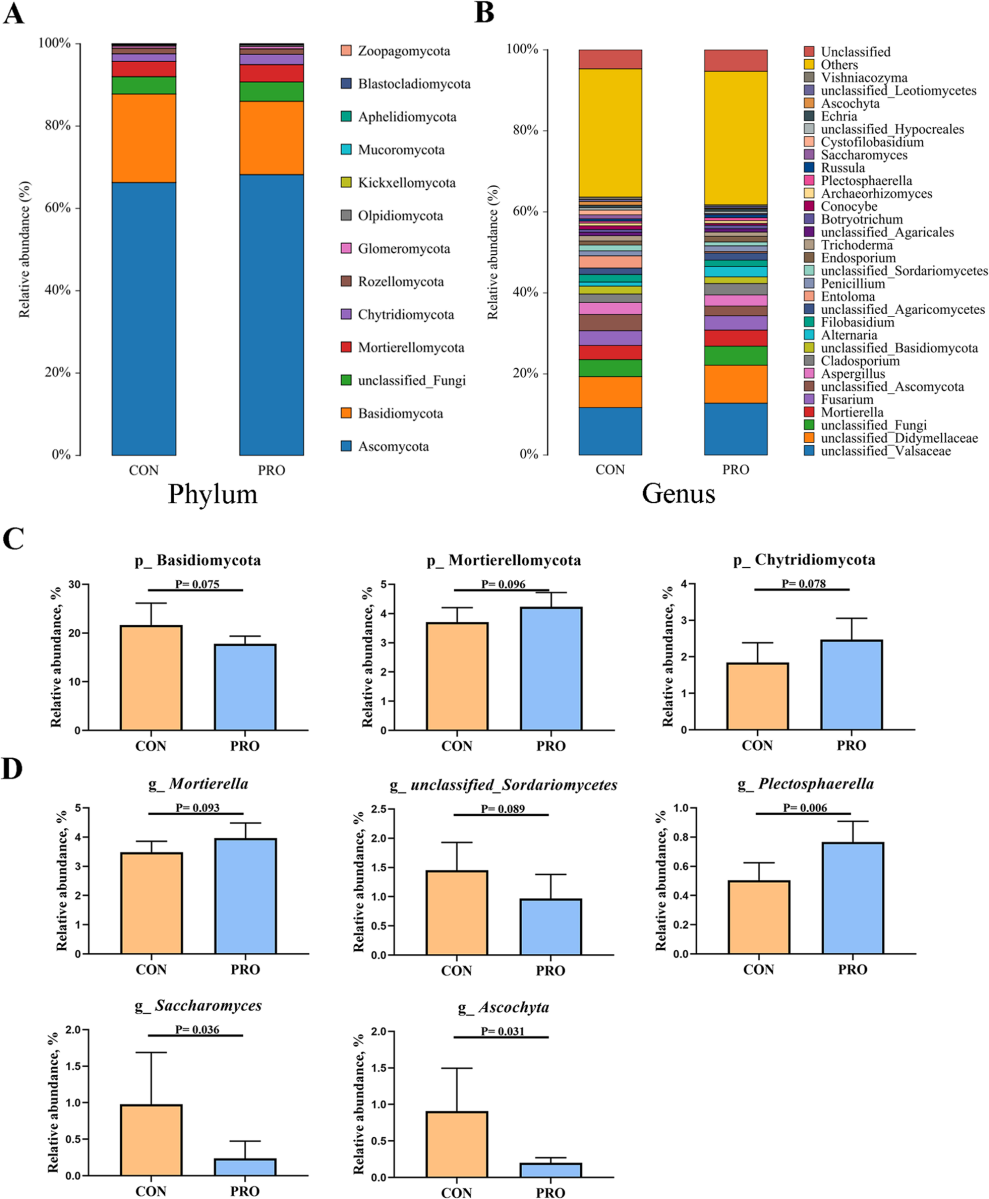
**A**, **B** Average relative abundance of fungus species in the feces at the phylum level (**A**) and genus level (**B**). **C**, **D** Relative abundance of fungus communities in the feces contents at the phylum level (**C**) and genus level (**D**). CON: Control group, PRO: Probiotics group. Data are expressed as mean ± SD (n = 6 per group)

The phylum of Ascomycota and Basidiomycota accounted for more than 80% of the total fungi abundance in pet cats. Compared with the control group, the abundance of Mortierellomycota and Chytridiomycota were increased and Basidiomycota was decreased in the probiotic group at the phylum level (*P* < 0.1) (Fig. 7A, C), while the abundance of Mortierella (*P* < 0.1) and Plectosphaerella (*P* < 0.05) was increased and the abundance of unclassified_ Sordariomycetes (*P* < 0.1), Ascochytahe (*P* < 0.05) and Saccharomyces (*P* < 0.05) was decreased in the probiotic group at the genus level (Fig. 7B, D).

### Compound Bacillus increased the levels of some fecal short-chain fatty acids in pet cats

**Fig. 8:**
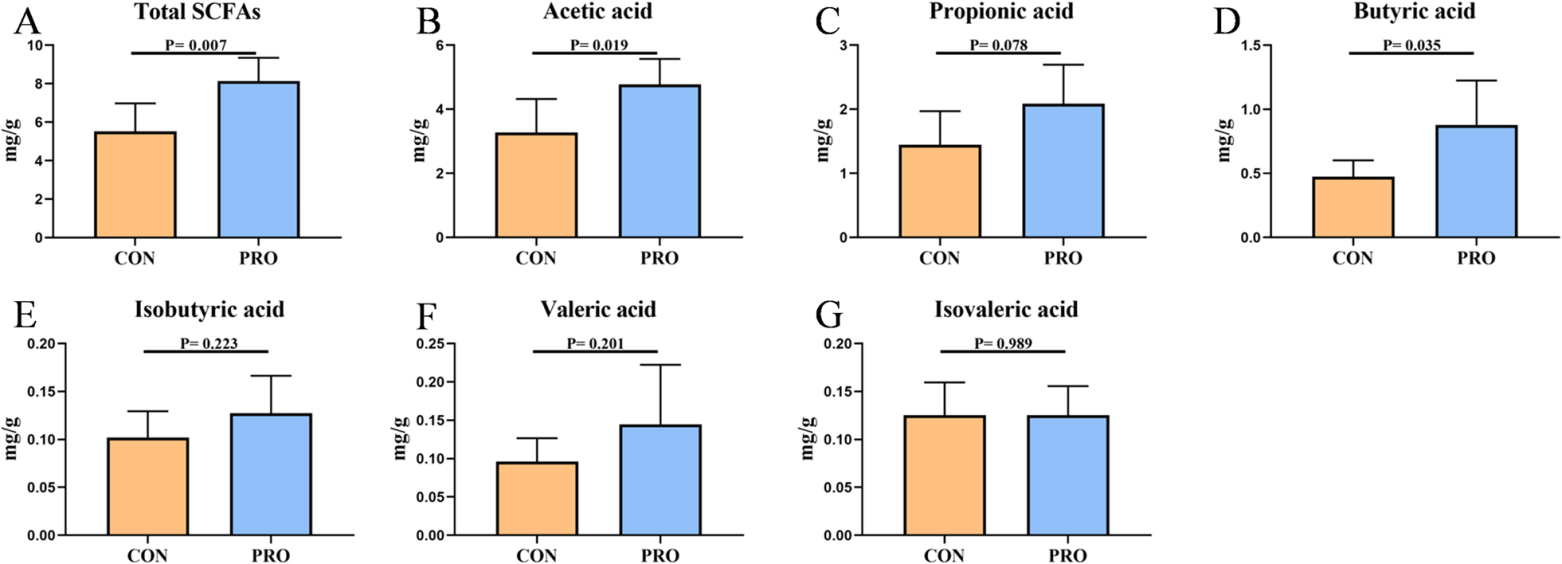
Effects of compound *Bacillus* on fecal Short-Chain Fatty Acids (SCFAs) in pet cats. **A** Total SCFAs, **B** Acetic acid, **C** Propionic acid, **D** Butyric acid, **E** Isobutyric acid, **F** Valeric acid, **G** Isovaleric acid. CON: Control group, PRO: Probiotics group. Data are expressed as mean ± SD (n = 6 per group)

Compared with the control group, the supplementation of probiotics significantly increased the contents of total SCFAs (CON: 5.12 mg/g vs. PRO: 8.14 mg/g, *P* < 0.05), acetic acid (CON: 3.28 mg/g vs. PRO: 4.78 mg/g, *P* < 0.05) and butyric acid (CON: 0.47 mg/g vs. PRO: 0.88 mg/g, *P* < 0.05), and the contents of propionic acid tended to increase (CON: 1.45 mg/g vs. PRO: 2.09 mg/g, *P* < 0.1), whereas there was no significant difference in isobutyric acid, valeric acid and isovaleric acid (*P* > 0.05) (Fig. 8).

### Fecal metabolic profiling analysis by untargeted HPLC/MS metabolomics

**Fig. 9:**
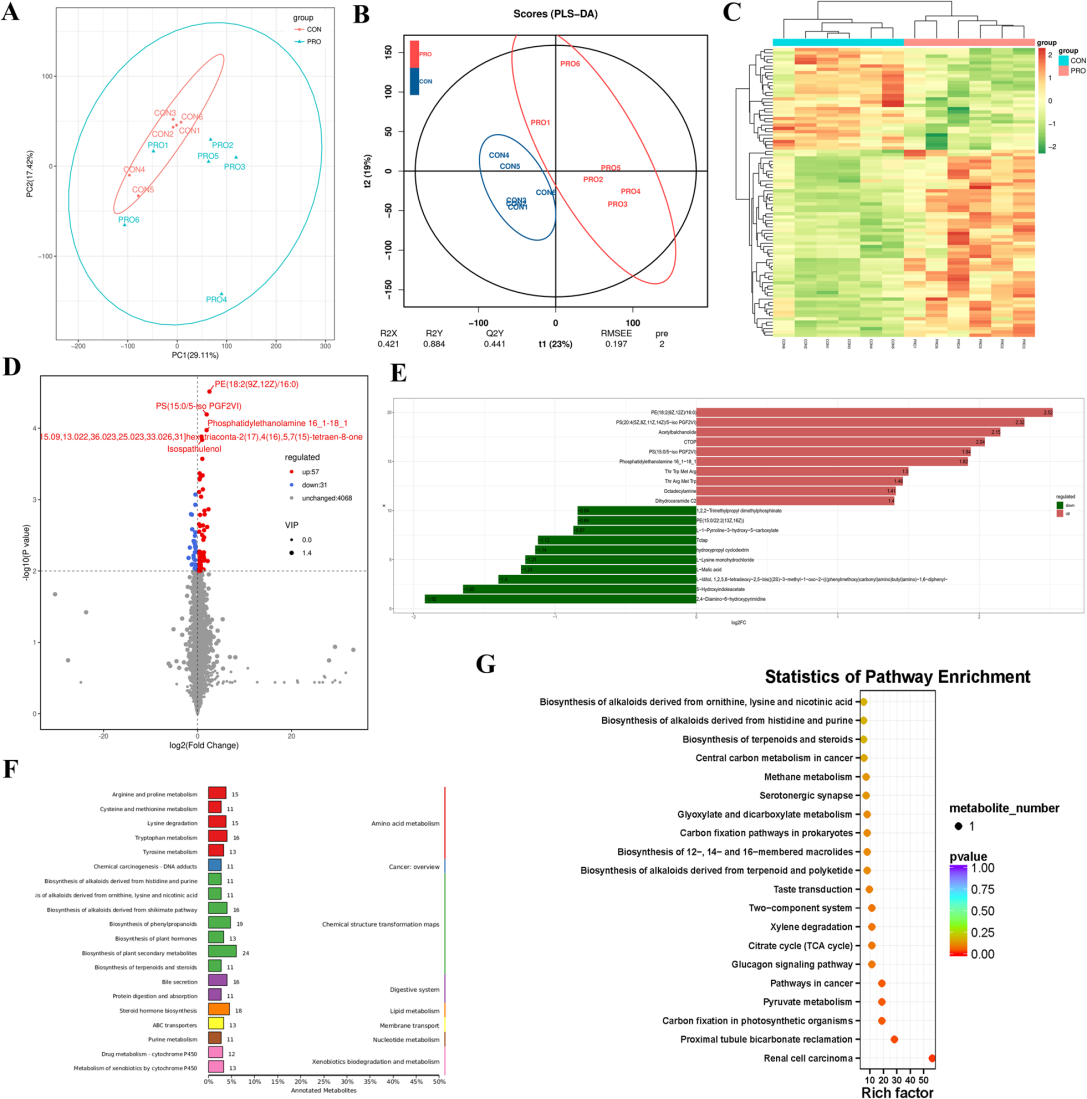
Multivariate statistical analysis (n = 6 per group). **A** Principal component analysis (PCA) score plot of nontargeted metabolite profiling of the fecal samples between two groups. **B** Score plots from the partial least-squares discriminant analysis (PLS-DA) model between two groups. **C** Hierarchical clustering analysis of fecal metabolites from CON and PRO groups. **D** Volcano graph of the distribution of the different metabolites in the CON and PRO groups, the red dots represent up-regulated metabolites and green dots represent down-regulated metabolites. **E** Top 20 FC-Change differential metabolites differential histogram. **F** KEGG pathway classification. **G** Bubble diagram showing the KEGG enrichment analysis. The bubble size indicates enriched numbers, while the color shade indicates the differences. CON: Control group, PRO: Probiotics group

Multivariate analyses were executed to reveal clustering trends between the two groups. The PCA was used to investigate the differences between the fecal metabolome of the two groups by the unsupervised statistical method. The PCA score plot showed that the separation between the two groups was not obvious (Fig. 9A). But the supervised pattern recognition of PLS-DA displayed class-discriminating variations. As shown in Fig. 9B, significant variations were obtained in the fecal metabolomes (component1 = 23%, component2 = 19%). The variation tendencies of fecal metabolites were displayed in a hierarchical clustering heatmap in Fig. 9C, suggesting significant variations between the two groups. In addition, different biological replicates also clustered together, indicating good homogeneity among biological replicates. A total of 4156 metabolites were detected by fecal metabolomics in this study. Based on the values of VIP > 1, $\left| {{\text{FC}}} \right|$ > 1 and *P* < 0.01, 88 metabolites were identified as potential biomarkers. The results showed that there were 88 differentially expressed metabolites between the two groups, among which 57 metabolites were up-regulated and 31 metabolites were down-regulated in the probiotic group (Fig. 9D). In the PRO group, the top ten fecal metabolites with significant increases were PE(18:2(9Z,12Z)/16:0), PS(20:4(5Z,8Z,11Z,14Z)/5-iso PGF2VI), acetylbalchanolide, CTOP, PS(15:0/5-iso PGF2VI), phosphatidylethanolamine 16_1-18_1, Thr Trp Met Arg, Thr Arg Met Trp, octadecylamine and dihydroceramide C2, and the most decreased metabolite was 2,4-Diamino-6-hydroxypyrimidine (Fig. 9E). KEGG enrichment analysis was employed to explore the most relevant pathways, revealed that most fecal compounds were involved in metabolism, particularly in chemical structure transformation maps and amino acid metabolism (Fig. 9F). The bubble diagram of KEGG signaling pathway shows that the phototropic pathway is mainly related to renal function, including renal cell carcinoma, proximal tubule bicarbonate reclamation and pyruvate metabolism. (Fig. 9G).

### Serum metabolic profiling analysis by untargeted HPLC/MS metabolomics

**Fig. 10:**
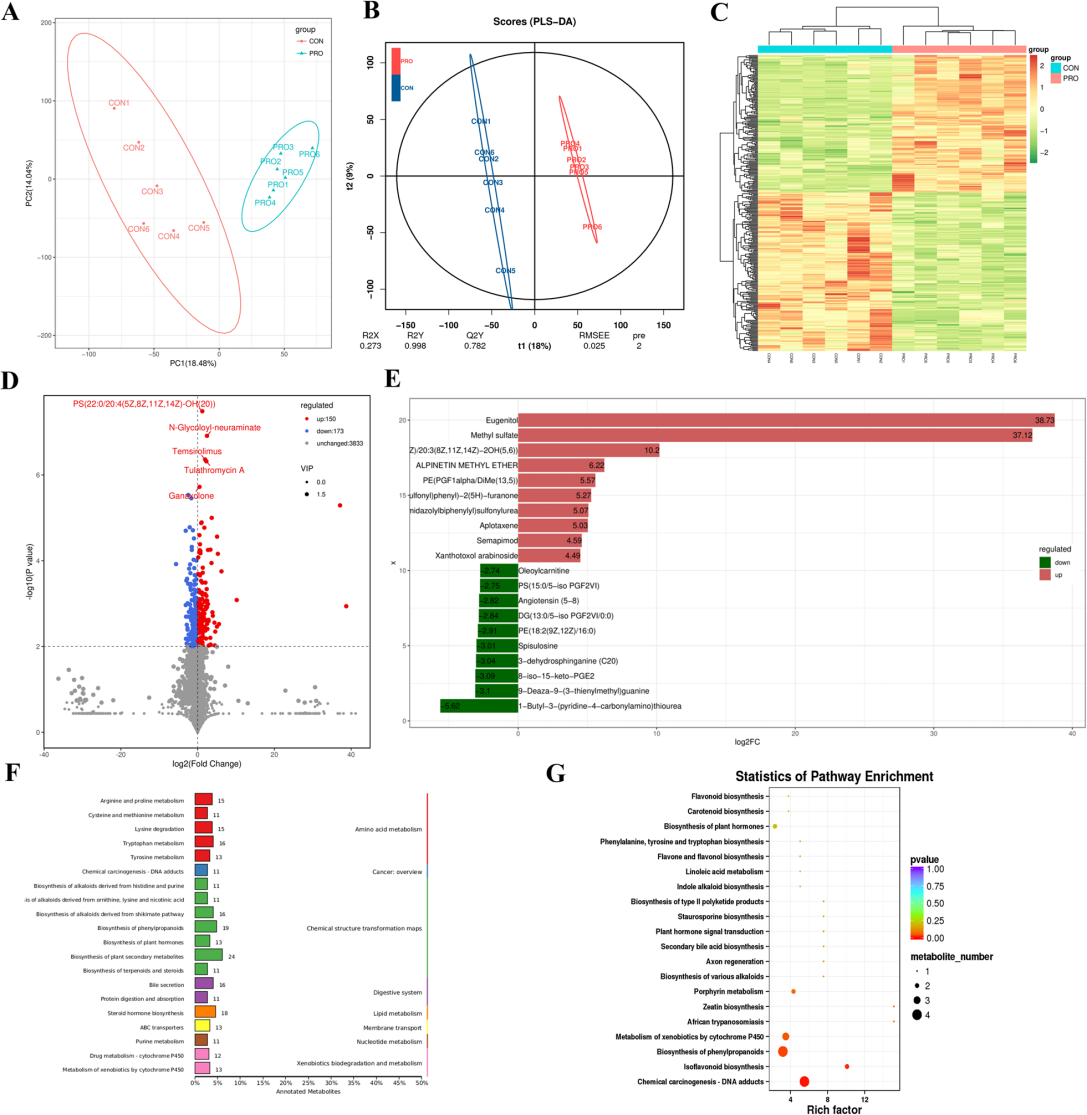
Multivariate statistical analysis (n = 6 per group). **A** Principal component analysis (PCA) score plot of nontargeted metabolite profiling of the serum samples between two groups. **B** Score plots from the partial least-squares discriminant analysis (PLS-DA) model between two groups. **C** Hierarchical clustering analysis of serum metabolites from CON and PRO groups. **D** Volcano graph of the distribution of the different metabolites in the CON and PRO groups, the red dots represent up-regulated metabolites and green dots represent down-regulated metabolites. **E** Top 20 FC-Change differential metabolites differential histogram. **F** KEGG pathway classification. **G** Bubble diagram showing the KEGG enrichment analysis. The bubble size indicates enriched numbers, while the color shade indicates the differences. CON: Control group, PRO: Probiotics group

Based on fecal metabolomics analysis, we further investigated serum metabolomics. As shown in Fig. 10A, B, the scores of both PCA (PC1 = 18.48%, PC2 = 14.04%) and PLS-DA models (component1 = 18%, component2 = 9%) showed obvious separation between the two groups after the addition of probiotics, indicating differences between the two groups. And like the fecal metabolome, the biological replicates in the two groups were also very homogenous (Fig. 10C). Similarly, a total of 4156 metabolites were detected in serum metabolomics, among which 323 metabolites were considered as potential biomarkers based on the values of VIP > 1, $\left| {{\text{FC}}} \right|$ > 1 and *P* < 0.01, including 150 up-regulated and 173 down-regulated metabolites (Fig. 10D). From the above analysis results showed that the differences of serum metabolites were greater than that of fecal metabolites. In the PRO group, eugenitol and methyl sulfate were the most significantly increased serum metabolites, and log_2_FC were 38.73 and 37.12, respectively. 1-Butyl-3-(pyridine-4-carbonylamino) thiourea, which log_2_FC was 5.62, was the most decreased metabolite (Fig. 10E). KEGG pathway classification revealed that most serum compounds were involved in metabolism, particularly in chemical structure transformation maps and amino acid metabolism, which the results were similar to those of the fecal metabolome (Fig. 10F). However, the KEGG bubble diagram showed significant differences in pathways related to biosynthesis, including isoflavonoid biosynthesis (number of metabolites = 2, *P *= 0.012) and biosynthesis of phenylpropanoids (4, *P *= 0.029) (Fig. 10G).

### Pearson’s correlation analysis

**Fig. 11:**
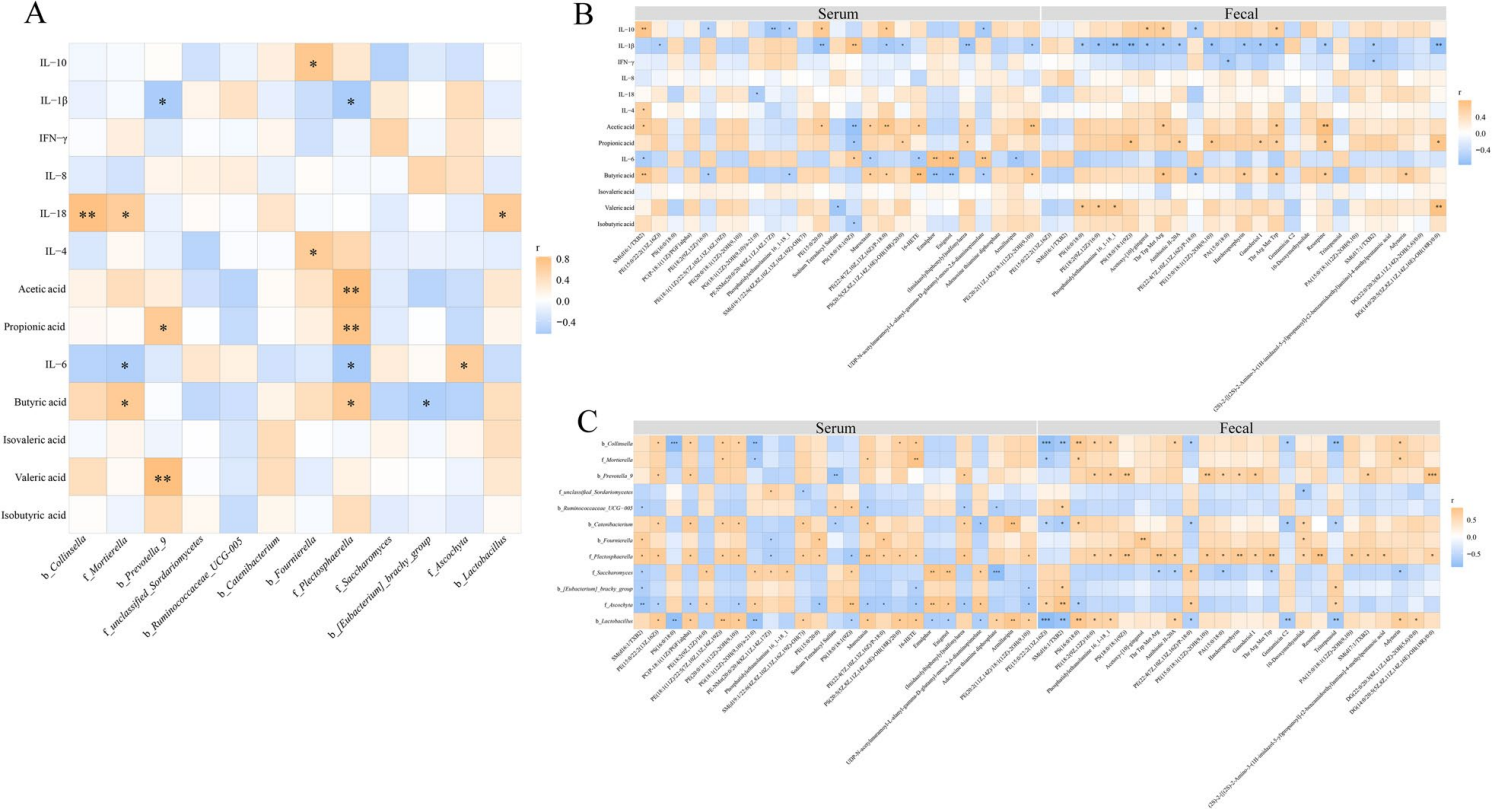
Heatmaps of Pearson’s correlation (n = 6 per group). **A** Correlation among the differential microbiota, immune parameters and SCFAs. **B** Correlation among the metabolic biomarkers, immune parameters and SCFAs. **C** Correlation between the differential microbiota and metabolic biomarkers. b_: bacteria, f_: fungus

**Fig. 12:**
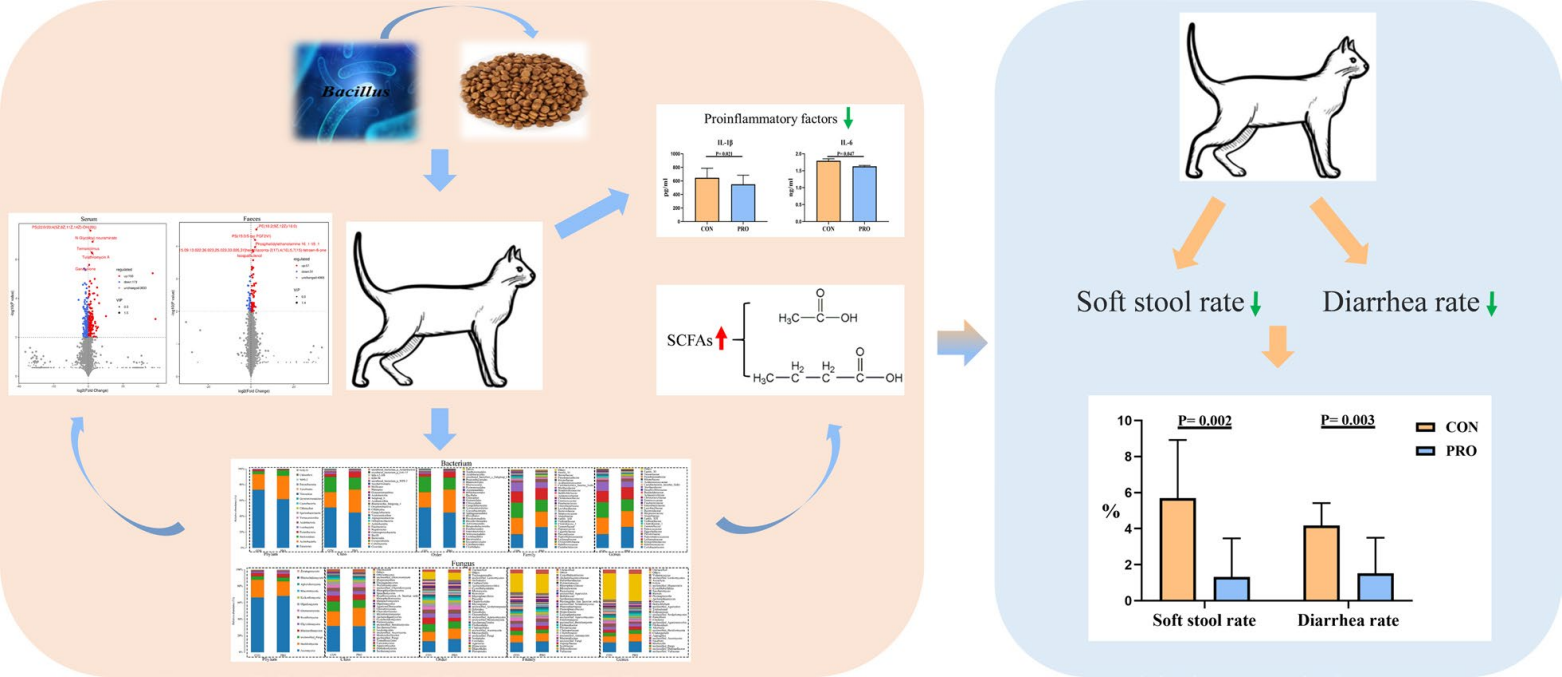
Graphical summary of compound *Bacillus* alleviates diarrhea by regulating gut microbes, metabolites, and inflammatory responses of pet cats

The correlations among metabolites in feces and serum, fecal microbes and indicators, which including immune functions and SCFAs, were examined to further confirm the underlying mechanisms. As shown in Fig. 11A, *Prevotella* was positively correlated with propionic acid and valeric acid, and negatively correlated with IL-1β, moreover, *Plectosphaerella* was positively correlated with acetic acid, propionic acid and butyric acid, and negatively correlated with IL-1β and IL-6. Figure 11B showed that IL-1β was negatively correlated with PE(15:0/22:2(13Z,16Z)), PE(15:0/20:0), PE(22:4(7Z,10Z,13Z,16Z)/P-18:0), PS(20:5(5Z,8Z,11Z,14Z,16E)-OH(18R)/20:0), (Imidazolylbiphenylyl)sulfonylurea and PE(20:2(11Z,14Z)/18:1(12Z)-2OH(9,10)) in serum, and PS(16:0/18:0), PE(18:2(9Z,12Z)/16:0), Phosphatidylethanolamine 16_1-18_1, PS(18:0/18:1(9Z)), Acetoxy-[10]-gingerol, Thr Trp Met Arg, Antibiotic JI-20A, PE(15:0/18:1(12Z)-2OH(9,10)), Harderoporphyrin, Ganoderiol I, Thr Arg Met Trp, Reserpine, SM(d17:1/TXB2) and DG(14:0/20:5(5Z,8Z,11Z,14Z,16E)-OH(18R)/0:0) in feces. PS(18:0/18:1(9Z)) was positively correlated with IL-1 and IL-6, but negatively correlated with acetic acid and propionic acid. In addition, Emulphor, Enigmol and UDP-N-acetylmuramoyl-L-alanyl-gamma-D-glutamyl-meso-2,6-diaminopimelate in feces were also significantly positively correlated with IL-6, while SM(d16:1/TXB2), Muroctasin, 16-HETE and Armillaripin in the feces were significantly negatively correlated with IL-6. SM(d16:1/TXB2), Muroctasin, PE(22:4(7Z,10Z,13Z,16Z)/P-18:0), 16-HETE and PE(20:2(11Z,14Z)/18:1(12Z)-2OH(9,10)) in the serum and Thr Trp Met Arg, Thr Arg Met Trp and Reserpine in the feces were positively correlated with acetic acid and butyric acid. Pearson’s correlation analysis was performed for the differential feces and serum metabolites and fecal microbiota (Fig. 11C), and results show that *Prevotella_9* was positively correlated with PS(18:0/18:1(9Z)), PE(15:0/18:1(12Z)-2OH(9,10)) and DG(14:0/20:5(5Z,8Z,11Z,14Z,16E)-OH(18R)/0:0) in the feces and negatively correlated with fecal Sodium Tetradecyl Sulfate. *Plectosphaerella* was strong positively correlated with Muroctasin in the serum and PS(18:0/18:1(9Z)), Thr Trp Met Arg, Harderoporphyrin, Thr Arg Met Trp and Reserpine in the feces (Fig. 12).

## Discussion

Diarrhea in pet cats is a serious and common problem in the daily life of pet cats, which will eventually affect the mental state and physical condition of pet cats. Due to the change of season and temperature, the change of living environment and diet, as well as the spread of bacteria, viruses and parasites, pet cats often have a stress response, which will affect the physical health of pet cats, may lead to diarrhea, loss of appetite, resistance, and mental collapse. Previous studies have shown that feeding *Enterococcus faecalis SF68* (2.1 × 10^9^ CFU/g) reduced diarrhea in cats compared with placebo [2]. In this study, we found that adding 3 × 10^9^ CFU/kg to the experimental diet effectively reduced the rate of soft stool and diarrhea in pet cats, which is consistent with previous findings.

Cytokines play a role in many fundamental processes of life and disease, including immunity, inflammation, embryonic development, regeneration, angiogenesis, metabolism, obesity, aging, and more [22]. As pro-inflammatory factors, IL-1β and IL-6 have been used to assess inflammatory and infectious responses and associated immunity in various animals, and they are associated with host defense, immune cell regulation, proliferation and differentiation [23–25]. Previous studies have shown that probiotics can significantly reduce the levels of IL-1β and IL-6 and increase the level of the anti-inflammatory cytokine IL-10 in the serum of mice with enteritis, thereby alleviating intestinal inflammation [26]. Similarly, our study showed that compound *Bacillus* supplementation also significantly reduced the levels of IL-1β and IL-6 in the serum of cats, and the level of IL-10 in the serum tended to increase.

Studies have proven that probiotics are related to the regulation of intestinal flora in preventing diarrhea and reducing inflammation [27]. Gut microbiota is a biological barrier against colonization by pathogenic bacteria, and its homeostasis plays a key role in animal health [28]. There were a large number of obligate anaerobic bacteria in cat feces, among which Firmicutes, Actinobacteria, Bacteroidetes, Proteobacteria, Fusobacteria and ranked in the top five in terms of abundance [29]. This study is consistent with it, but interestingly, the abundance of Firmicutes in the probiotic group is significantly lower than that in the control group. The Firmicutes include inflammation-associated microorganisms like *Clostridium*, *Lachnospiraceae*, and *Erysipelotrichaceae*, whereas Bacteroidetes include more health-promoting members like *Ruminococcaceae*, *Bifidobacterium*, and *Lactobacillus* [30]. The Firmicutes/Bacteroidetes ratio was used to determine the health state of the host [31]. Previous studies have shown that the increase of the Firmicutes/Bacteroidetes ratio may lead to obesity and autism spectrum disorders [32, 33], our study shows that probiotics can prevent this from happening. And results were further verified with LEfSe analysis, which also found that the control group was associated with enrichment of *Sutterella*, *Turicibacter*, and *Enterococcus*, while *Lactobacillus* dominated the probiotics treatment. *Sutterella* belongs to the phylum Proteobacteria, which over secretes IgA protease and degrades IgA, thus reducing the IgA concentration in intestinal mucosa and damaging the function of intestinal antimicrobial immune response [34]. In addition, *Sutterella* is one of the important sources of LPS, which can affect intestinal permeability, and has a positive correlation with diarrhea [35]. *Turicibacter* may play an important role in neurotransmitter system dysfunction in autism and may continuously disrupt neurotransmitter system homeostasis, mainly through interference with the 5HT system [36]. However, cats fed with compound *Bacillus* apparently stopped this trend and increased the abundance of *Lactobacillus*, which contribute significantly to nutrient absorption and related intestinal function by participating in the synthesis of some essential vitamins and organic acids [37].

In addition to bacteria, fungi also play an important role in intestinal flora. Fungi are a large group in the biological world, as a crucial component of the microbial community. Previous sequencing results have shown that Basidiomycota, Chytridiomycota and Ascomycota were the dominant species in the GI tract of animals [38]. Our study shows that Ascomycota and Basidiomycota in cat feces accounted for about 80% of fungal abundance at the phylum level. Due to the large number of diverse structured chemical compounds they produce, fungi from the phyla of Ascomycota, Basidiomycota and Muccoromycota have been intensively studied for isolation of bioactive compounds, which are known as a promising source of antibacterial compounds with activity against Gram-positive bacteria [39]. Interestingly, in this study, we found that the abundance of *Plectosphaerella* could be increased after the addition of probiotics. As early as 1929, Klebahn came up with *Plectosphaerella* and isolated it from cucumber to get *Plectosphaerella cucumerina*. Pearson’s correlation analysis showed that *Plectosphaerella* were significantly positively correlated with short-chain fatty acids (acetic acid, propionic acid and butyric acid), and were negatively correlated with pro-inflammatory factors (IL-1β and IL-6). These results indicate that *Plectosphaerella* can produce short-chain fatty acids and reduce the level of inflammatory factors, but the exact conclusion and mechanism need further investigation.

The level of SCFAs is closely related to the microbial structure [40]. Different florae produce different SCFAs, which have been reported to protect the gut by providing energy and creating a low pH environment to inhibit pathogen growth. Lack of SCFAs attenuates its protective effect on the intestinal mucosal barrier, leading to increased endogenous endotoxin. In addition, SCFAs can regulate the production, trafficking, and function of innate and adaptive immune cells [41, 42]. Acetic acid is an important factor in regulating pH, thus maintaining the acidic environment in the intestinal lumen [43]. In addition, acetic acid and propionic acid can regulate the production of inflammatory mediators and enhance the phagocytosis of infectious cells by immune cells [44]. Butyrate, a type of SCFAs, can directly act on immune cells in the intestinal mucosa, increase the number and activity of Tregs (Regulatory T cells), and inhibit the activity of neutrophils, macrophages, dendritic cells and effector T cells [45]. Our study showed that probiotics could significantly increase the contents of acetic acid and butyric acid in the feces of pet cats, which may be one of the important factors to reduce the rate of soft stool and diarrhea in pet cats.

Microbial derivatives are important regulators of host metabolism and play an important role as they are secreted in the gut and cross the intestinal barrier into the circulatory system [46]. The results of the fecal metabolite group showed that compared with the control group, the content of metabolite PE(18:2(9Z,12Z)/16:0) in the probiotics group increased the most, which is a kind of glycerophosphoethanolamines. Phosphoethanolamine (PE) is a class of phospholipids found in biological membranes and is involved in the formation of most biofilm scaffolders [47]. Phosphatidylserine (PS) is an endogenous phospholipids, which has been reported to exert an anti-inflammatory activity by downregulating pro-inflammatory molecules [48]. In this study, the compound *Bacillus* has a significant effect on the metabolism of pet cats at both fecal and serum levels. The most interesting thing is that the content of eugenitol and methyl sulfate in serum metabolites of pet cats fed with compound *Bacillus* increased significantly compared with the control group, but no similar pattern was found in fecal metabolomics. Eugenitol has a similar structure with biflorin and noreugeninshare, which have common structures such as methylchromone with two hydroxyl groups, and they had been reported to exert anti-inflammatory effects [49, 50]. In addition, studies have further shown that eugenitol had the effects of anti-inflammatory and antioxidants through LPS-induced BV2 cell activation experiments [51]. A previous study showed that hydroxycinnamates were preferentially hydrolyzed and subsequently methylated, being mostly conjugated to sulfate, methyl- and methyl-sulfate derivatives [52], indicating that methyl sulfate is involved in organism metabolism and has a positive effect. However, at present, there are few relevant functional studies on eugenitol and methyl sulfate, which their specific mechanism of action is not yet clear and relevant experiments can be conducted to further explore the mechanism in the later stage. In this study, correlation analysis showed that PS (16:0/18:0) and PS (18:0/18:1(9Z)) in the feces were negatively correlated with IL-1β, and positively correlated with valeric acid and propionic acid, respectively. These results can be interpreted as suggesting that compound *Bacillus* can maintain phospholipid homeostasis and reduce inflammation by increasing PE and PS levels in the gut. KEGG enrichment analysis of fecal metabolites showed changes in some renal and urinary pathways, such as renal cell carcinoma and proximal tubule bicarbonate reclamation, so we can take further experiments to investigate whether compound *Bacillus* have effects on the urinary tract and kidney of pet cats in the future.

## Conclusion

In conclusion, dietary supplementation of compound *Bacillus* can increase the abundance of p_ Patescibacter as well as g_ *Plectosphaerella*, and decreased the abundance of p_ Firmicutes, p_ Gemmatimonadetes, g_ *Ruminococcaceae_UCG-005*, g_ *Ascochytahe* as well as g_ *Saccharomyces*. Moreover, it also was increased the contents of eugenitol and methyl sulfate in the serum, as well as total SCFAs, acetic acid and butyric acid in the stool, decreased the contents of IL-1β and IL-6 in the serum, which thus relieving diarrhea in pet cats.

### Supplementary Information

**Additional file 1: Table S1.** Ingredients and nutrient contents of the basal diet (DM basis). **Table S2**. Scoring standard of fecal score.

## Data Availability

The datasets produced and/or analyzed during the current study are available from the corresponding author on reasonable request.

## Abbreviations

FS: Fecal scores
IL: Interleukin
TNF-α: Tumor necrosis factor-α
PS: Phosphatidylserine
PE: Phosphoethanolamine
