# Supplementary material for: Compound Bacillus alleviates diarrhea by regulating gut microbes, metabolites, and inflammatory responses in pet cats
Source: Anim Microbiome. 2023 Oct 10;5:49. doi: 10.1186/s42523-023-00270-8 (PMC10566145; doi:10.1186/s42523-023-00270-8)
Supplement: Supplementary file 1 — Additional file 1: Table S1. Ingredients and nutrient contents of the basal diet (DM basis). Table S2. Scoring standard of fecal score. [file 42523_2023_270_MOESM1_ESM.docx]

Supplemental materials

Table S1 Ingredients and nutrient contents of the basal diet (DM basis)

| Ingredients | Content, % | Nutrient ^b^ | Content, % |
| --- | --- | --- | --- |
| Chicken | 30.05 | Crude protein | 42.00 |
| Beef powder | 20.60 | Ether Extract | 23.80 |
| Frozen beef | 8.58 | Crude fiber | 2.20 |
| Frozen fish | 8.58 | Moisture | 5.30 |
| Duck meat powder | 5.15 | Ash | 7.60 |
| Fish meal | 4.29 | Calcium | 1.43 |
| Sweet potato powder | 4.27 | Total phosphorus | 1.27 |
| Potato powder | 4.27 | Water-soluble chloride | 0.75 |
| Chicken fat | 3.88 | Taurine | 0.58 |
| Fish oil | 3.88 |  |  |
| Alfalfa particles | 1.72 |  |  |
| Chicken liver powder | 1.72 |  |  |
| Carrot | 0.43 |  |  |
| Egg powder | 0.43 |  |  |
| Chlorella powder | 0.43 |  |  |
| Blueberry | 0.08 |  |  |
| Beer yeast | 0.08 |  |  |
| Additive ^a^ | 1.56 |  |  |
| Total | 100.00 |  |  |

^a^ The premix provided the following per kg of the diet: Fe, 390 mg; Mn, 27 mg; Cu, 16 mg; Zn, 110 mg; I, 4.53 mg; Se, 0.65 mg; VA, 35 500 IU; VD, 32 660 IU; VE, 59.8 IU; VK, 0.728 mg; VB, 17.88 mg; VB, 212.9 mg; pantothenic acid, 47 mg; nicotinic acid, 67.6 mg; pyridoxine, 12.3 mg; biotin, 0.14 mg; folic acid, 0.8 mg.

^b^ Nutrient levels were measured values.

Table S2 Scoring standard of fecal score

| Score | Characteristics |
| --- | --- |
| 1 | - Very hard and dry. - Expelled as individual pellets. - Leaves no residue on the ground when picked up. |
| 2 | - Solid but not hard. - Segmented in appearance. - Leaves little or no residue on the ground when picked up. |
| 3 | - Log shape, wet surface. - Little or no visible segments. - Leaves residue on the ground and deformation when picked up. |
| 4 | - Very wet. - Present in piles rather than logs. - Leaves residue on the ground and distortion when picked up |
| 5 | - No definite shape. - Appears as speckled or liquid. - Can’t pick up. |

1 ≤ FS < 2 is constipation, 2 ≤ FS ≤ 3 is normal, 3 < FS < 4 is soft stool, 4≤FS≤5 is diarrhea.
